# Unconscious response priming during continuous flash suppression

**DOI:** 10.1371/journal.pone.0192201

**Published:** 2018-02-05

**Authors:** Mika Koivisto, Simone Grassini

**Affiliations:** Department of Psychology, University of Turku, Turku, Finland; University of Valencia, SPAIN

## Abstract

Continuous flash suppression (CFS) has become a popular tool for studying unconscious processing, but the level at which unconscious processing of visual stimuli occurs under CFS is not clear. Response priming is a robust and well-understood phenomenon, in which the prime stimulus facilitates overt responses to targets if the prime and target are associated with the same response. We used CFS to study unconscious response priming of shape: arrows with left or right orientation served as primes and targets. The prime was presented near the limen of consciousness and each trial was followed by subjective rating of visibility and a forced-choice response concerning the orientation of the prime in counterbalanced order. In trials without any reported awareness of the presence of the prime, discrimination of the prime’s orientation was at chance level. However, priming was elicited in such unconscious trials. Unconscious priming was not influenced by the prime-target onset-asynchrony (SOA)/prime duration, whereas conscious processing, as indicated by the enhanced discriminability of the prime’s orientation and conscious priming, increased at the longest SOAs/prime durations. These results show that conscious and unconscious processes can be dissociated with CFS and that CFS-masking does not completely suppress unconscious visual processing of shape.

## Introduction

Continuous flash suppression (CFS) [1] is an inter-ocular technique that has become a popular tool for suppressing visibility of a stimulus and studying unconscious processing. In CFS, the stimulus is presented to one of the eyes, while a strong, high contrast mask is flashed at 10–20 Hz to the opposite eye. In this way, the stimulus may remain invisible for several seconds [2]. The recent enthusiasm for CFS-masking is largely based on this long suppression time, which is considerably longer than the 10–40 ms that is obtained with traditional forms of masking, such as backward masking by metacontrast or pattern mask [3]. Thus, the traditional masking procedures can provide only a temporally limited input for the visual system. Therefore, it is possible that complex unconscious processes that can be potentially performed by the cognitive system, but which may require a longer visual input duration, may be unreachable with the traditional methods but can be studied with CFS. Indeed, recent CFS studies have suggested that unconscious processes are able to perform rather complex tasks, such as integration of an object with a semantically congruent scene [4, but see 5] or processing multiple word expressions and solving arithmetic equations [6].

CFS is used to study unconscious cognitive processes in the form of breaking-CFS (b-CFS) or unconscious priming. Here we focus on unconscious priming, in which the visibility of a prime stimulus is suppressed by CFS, and its influence on responding to the target stimulus is measured. The results concerning the level at which unconscious primes can elicit priming have been mixed in CFS studies. Bahrami et al. [7] found unconscious numerical priming during CFS, but recent replication attempts with more appropriate baseline measures found only low-level repetition priming but not any unconscious numerosity priming [8, 9]. Barbot and Koudier [10] reported unconscious repetition priming with neutral face images, with the facilitation effect reversing to inhibitory effect (negative priming) at long prime durations. Gelbard-Sagiv et al. [11] also studied repetition priming of faces with a long prime duration, but priming (inhibitory effect) occurred only when a low-level visual feature of the prime (color or location) was accessible to consciousness. Semantic priming, as measured with the N400 effect in event-related potentials, did not occur under CFS for word stimuli [12], but another study found evidence for semantic processing by showing that suppressed words facilitated problem solving [13]. Some of the priming experiments with emotional faces have reported that suppressed emotional faces do not bias subsequent preference judgments, although low-level repetition priming can be observed [14], but others have found that preference judgments are biased by suppressed emotional faces [15, 16] and that unconsciously processed facial expressions can influence the conscious recognition of facial expressions [17].

A series of studies [18, 19] found priming after pictures of tools but not after pictures of animals, when the primes were presented during CFS with high spatial-frequency visual noise serving as mask. During backward masking, both tool and animal primes elicited unconscious priming. According to Almeida et al.’s interpretation, tools were processed by the “vision-for-action” dorsal stream [20] and CFS left its functioning relatively intact while interfering more with processing of the “vision-for-perception” ventral stream. The interpretation that the tools specifically escaped masking was questioned by a CFS study [21] which found that it was the elongated shape of objects (i.e., tool pictures) that determined whether priming occurred or not. A recent study [22] found unconscious priming for elongated objects only when the task was to make decisions on the basis of the shape, but no priming for the same stimuli when the decisions were made regarding the category of the stimuli (tool vs. animal). Another recent study [23] found that CFS-masked images of animals (e.g., dog) produced priming of superordinate category level decisions (animal vs. non-animal), but not of basic level decisions (e.g., dog vs. bird). It cannot be ruled out that priming of superordinate level was based on shape or other low level features rather than on category *per se*. Thus, the results from studies on unconscious priming during CFS do not paint a coherent picture of the processes that survive CFS: there is no agreement on whether only low level processing occurs during CFS or whether high level semantic processes can survive CFS.

Response priming is a robust phenomenon and well-suited for exploring early visuomotor processing in a wide range of tasks and research fields [24]. In response priming, responses to the targets are facilitated if the preceding prime is associated with the same response as the target (e.g., “left” to left-oriented prime and target arrows), whereas the responses are slowed down if an incongruent response is associated with the prime [24–26]. Many of the priming studies cited above [18,19, 21–23] used variants of response priming to study unconscious processing. They indeed found unconscious priming under CFS, although it seems that it may be limited to priming of shape. However, a recent study by Peremen and Lamy [27] did not find unconscious response priming of arrow shapes during CFS, although priming was observed when primes were masked by metacontrast.

In Peremen and Lamy [27], the contrast level of the prime was near the threshold for consciousness so that in about 50% of the trials the presence of the prime was consciously detected. The conscious visibility of the primes was measured in trial-by-trial manner with a graded Perceptual Awareness Scale (PAS) [28] and only the trials in which the participants reported not seeing any prime at all were included in the analyses of unconscious priming. This procedure guaranteed that the primes were not so strongly masked that any perceptual processing, conscious or unconscious, would have been suppressed. It was concluded that when the prime is completely suppressed from awareness by CFS, response priming does not occur [27]. Nonetheless, this conclusion stands in contrast to the findings showing that shapes elicit unconscious priming during CFS [21,22].

There are several technical aspects in Peremen and Lamy’s [27] procedure that may not have been optimal for obtaining unconscious response priming during CFS. First, the stimulus-onset asynchronies between the prime and target (SOAs), which corresponded also to the duration of the prime, may have been too long (250, 350, 450, 550, and 650 ms in Experiment 2, and 200 ms in Experiment 3). If response priming is triggered by the onset of the prime, the priming effect may have been faded away at the onset of the target due to the long SOAs. Second, the contrast of the prime was ramped up from 0% to 20%, 60%, or 100% in 200 ms (Experiment 2) or from 0% to 100% in 50 ms (Experiment 3). As both invisible unconsciously processed primes and visible consciously processed primes produce contrast-dependent priming [29], the gradual ramping up of the prime’s contrast may have influenced the priming effects by eliminating the fast, transient signal that may drive response priming. The gradual ramping up of the contrast is usually employed in b-CFS paradigm [4,5], not when CFS is used to mask the visibility of primes [18, 19, 21–23, but see 11]. In addition, the overall speed of responding was rather slow (> 680 ms), while typically the response times in corresponding tasks requiring judgements on the orientation of the target are 300–400 ms [26]. Assuming priming effects are short-lived, it is possible that the gradual onset of the primes and the slow response speed may not have been optimal for measuring small, short-lived effects.

We aimed to reduce the confusion surrounding the presence or absence of unconscious priming of shapes under CFS and to show that with a different procedure unconscious response priming effect for arrow shapes can be elicited by CFS masked primes. While in Peremen and Lamy [27] the shortest SOA was 200 ms and the onset of the prime was ramped up, we used SOAs of 94, 188, or 282 ms with abrupt prime onset. Peremen and Lamy [27] measured the visibility of the primes in each trial only with a subjective PAS scale [28]; we used in each trial also a 2-alternative forced-choice decision task on the orientation of the prime, so that we could verify the validity of the subjective scale as a measure of consciousness during exactly the same trials in the priming task. As subjective visibility of the primes was measured in trial-by-trial manner during the priming task, attention was divided between targets and primes. Therefore, we did not use the approach in which conscious visibility and/or prime discrimination is assessed in a separate control task, because in such a task attention would be fully focused on the prime, which overestimates the occurrence of conscious prime perception as compared with the divided-attention condition during the priming task [30]. The divided-attention task is more balanced in this respect: conscious perception and discrimination of the prime is measured in exactly the same multi-task condition in which priming is measured. We calibrated the contrast for each participant individually such that about 50% of the primes, or slightly less, would be rated as completely invisible under CFS. Under such presentation condition, we expect on basis of our earlier experience [31] that the participants would never or very rarely see the prime clearly and they would use predominantly the ‘saw nothing’ and ‘saw something’ alternatives. Therefore, we modified the subjective scale to be more consistent with participants’ experiences. Instead of the 4-point subjective scale [27], we used only three points in the subjective scale (‘saw nothing’, ‘saw a glimpse of something’ and ‘I think/guess I saw the prime’s orientation’; the last one replacing the ‘almost clear’ and ‘clear’ ratings in the original PAS scale).

The response-relevant characters of the mask may bias the responses to the target stimuli [32]. For example, if the mask in a response priming task with arrow stimuli contains arrowhead-like elements, these response-relevant elements may activate responses that are associated to them, thus disrupting the priming process. The masks in Peremen and Lamy [see Fig 5 in ref. 27] included shapes that resembled arrowheads, which may have biased participants’ left-right responses to the targets (i.e., arrows) and disrupted unconscious priming by overriding the activations generated by the primes. We designed our Mondrian masks in such way that they consisted only of squares and rectangles, avoiding possible directional cues.

If response priming will be observed in trials without reported awareness of the prime (‘saw nothing’ ratings) and without better than chance performance in the forced-choice prime discrimination task, the results will provide evidence for unconscious processing of shape under interocular suppression.

## Methods

### Participants

Sixteen students from the University of Turku volunteered (age 20–29 years, 6 males) to fulfill a partial course requirement in introductory psychology. They had a normal or corrected-to-normal binocular vision. The study was conducted in accordance with the Declaration of Helsinki and with informed and written consent of each participant. The study was approved by Ethics Committee of Hospital District of Southwest Finland (ETMK 138/2013, 26.11.2013 § 388).

### Stimuli and procedure

**Fig 1:**
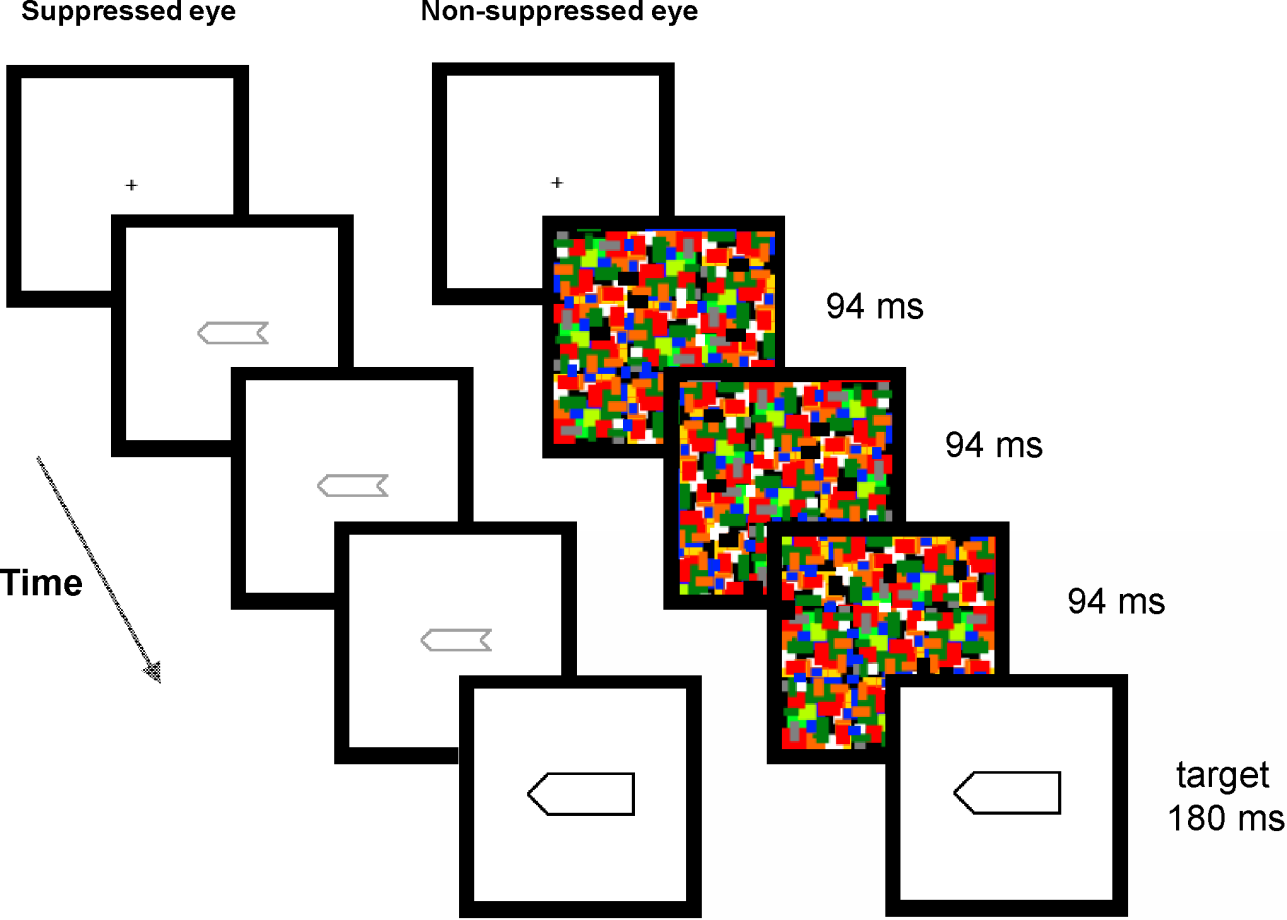
Stimuli and procedure. The sequence of stimulation in the 282 ms stimulus-onset asynchrony/prime duration condition. The participants responded to the orientation of the target and then rated the visibility of the prime and made a forced-choice response to the orientation of the prime.

The prime stimuli were left and right oriented arrows (2.9 x 1.0°)(Fig 1). The targets were left and right oriented arrows which were larger than the primes (4.5 x 1.8°). When the prime and target were superimposed, the outer contours of the prime were separated by 0.3° from the inner contours of the target. The experiment used as masks Mondrians consisting of colorful, overlapping rectangles (0.7° x 0.4°; range: 0.2–1.0). The luminance of the rectangles in the masks varied from 91 cd/m^2^ (white) to 5 cd/m^2^ (black).

The stimuli were presented on a 19” CRT monitor with 1048 x 786 pixels resolution at 85 Hz screen refresh rate on a white background (91 cd/m2). The monitor was viewed through a mirror stereoscope at the distance of 30 cm. To facilitate binocular fusion, the stimuli and masks were surrounded by black frames (8.9 x 8.9°) which were presented to each eye.

After the fixation point in the center of the screen in both eyes (Fig 1), the prime was presented to one eye for 94, 188, or 282 ms (except only for 94 ms in unmasked condition; catch trials did not involve any prime but blank screen instead of it), followed immediately by the target into both eyes for 180 ms. The three prime durations and the eye receiving the prime were randomized. The masking started simultaneously with the appearance of the prime. The mask was flashed to the opposite eye relative to that of the prime and it altered at 11 Hz (i.e., every 94 ms). The experimental trials were run in four blocks. The experiment involved 704 trials for each participant: 192 critical masked trials at each of the three SOAs/prime durations (half congruent and half incongruent), 32 catch trials at each SOA/prime duration, and 32 unmasked trials.

The participants were asked to make a manual left/right decision concerning the orientation of the target as quickly and accurately as they could. This was indicated by pressing the top buttons no. 5 or 6 in Logitech Dual Action gamepad with their left or right middle fingers. After responding to the target, two judgements were given. The participants made a non-speeded forced-choice decision concerning the left-right orientation of the prime and consciousness rating (i.e., they rated the visibility of the prime [28]). The left-right forced-choice responses to the prime’s orientation were given with the left and right middle fingers by pressing buttons (no. 7 or 8) which were located below the buttons (no. 5 and 6) used for responding to the target. The rating response was given with the four buttons located on the right side of the pad, below the right thumb; honesty and accuracy of the rating was stressed. The rating alternatives were ‘I saw nothing’ (button at 0 o’clock), ‘I saw a glimpse of something’ (button at 3 o’clock), and ‘I think/guess I saw the orientation’ (button at 6 o’clock). It was stressed that if they had any kind of perception or feeling about the orientation of the prime, they should use the last alternative. To help the participants to attend to the location of the prime, they were also informed that the prime always appeared inside the area that was surrounded by the inner contours of the target.

The contrast of the prime was determined for each participant individually on basis of their ability to detect the presence of the prime (i.e., rating other than ‘I saw nothing’) in a calibration block performed before the experimental trials. The procedure was the same as in the experimental blocks described above, with the exception that the contrast of the prime was varied (-0.67, -0.56, -0.45, or -0.34 Weber contrast). The contrast level at which the prime detection proportion was near 50% or slightly below (averaged across the SOAs/ prime durations) was selected. The calibration block was preceded by a practice block and followed by the experimental blocks. The calibration block consisted of 120 trials, involving 96 masked trials (half congruent, half incongruent; 24 trials at each four contrast levels of the prime), 12 catch trials, and 12 unmasked trials. The catch trials did not include any prime and their length corresponded to either 94, 188, or 282 ms SOA/prime duration conditions.

## Results

### Consciousness

**Fig 2:**
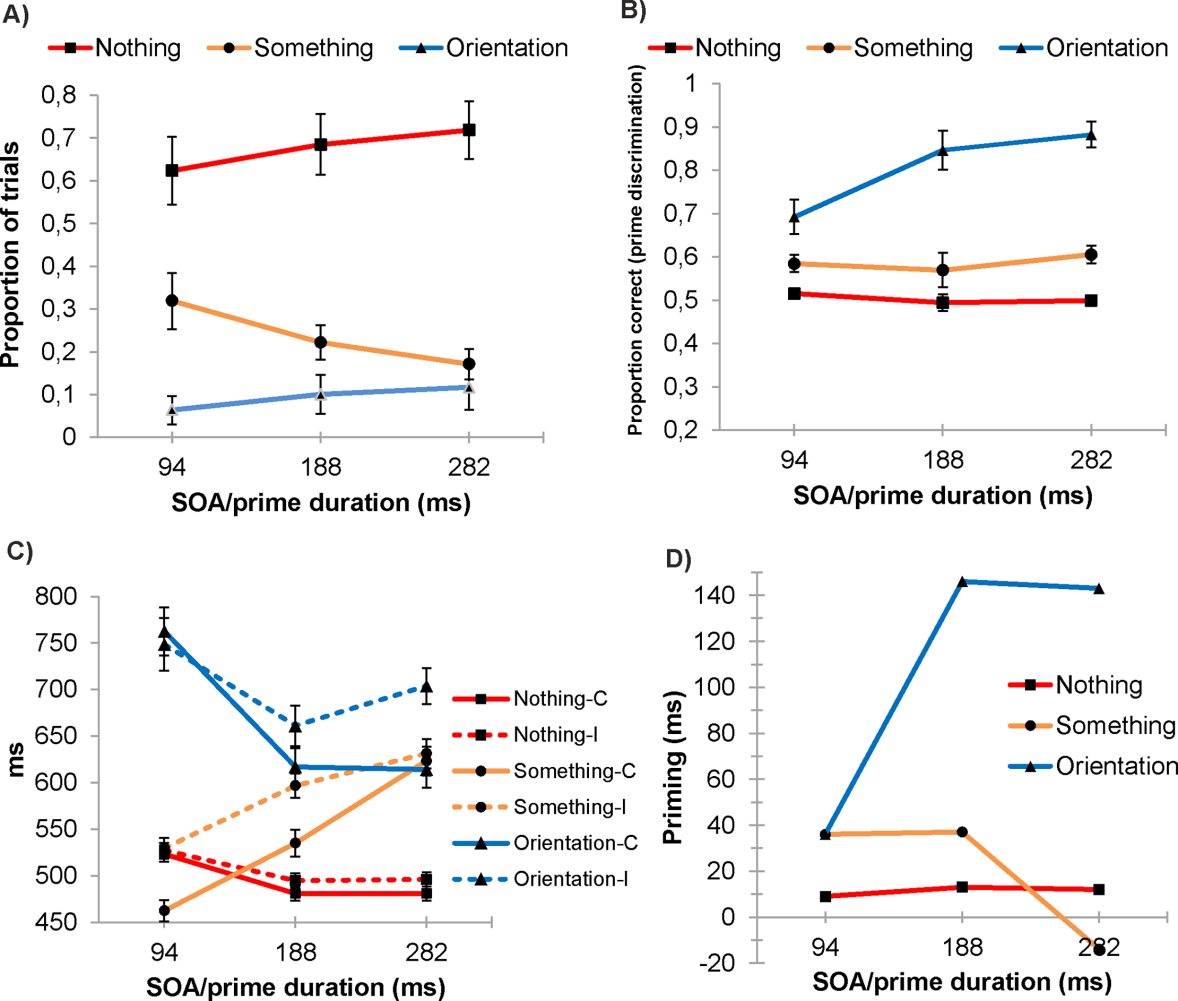
Results. (A) Rated consciousness of the prime in the critical trials as a function of SOA/prime duration. The participants reported that they either did not see anything presented (= nothing), they saw a glimpse of something (= something), or they think they saw or could guess the orientation of the prime (= orientation). (B) Accuracy of the two-alternative forced-choice responses to the orientation of the prime. (C) Observed response times to congruent (c; solid line) and incongruent (i; dotted line) targets, and (D) modeled priming effects. Error bars represent SEM.

For the critical CFS masked trials (Fig 2A), the Consciousness (3) x SOA/prime duration (3) ANOVA on the frequency of consciousness ratings showed a main effect for Consciousness, *F*(1.29,19.39) = 24.74, *p* < 0.001, η^2^_p_ = .62, indicating the that in most of the trials the participants did not have any conscious perception of the presence of the prime (rating ‘saw nothing’, 67.6%, *SE* = 6.6). They reported having seen nothing more frequently than having seen a glimpse of ‘something’ (23.7%, *SE* = 3.9)(*p* = 0.001) or having seen the prime’s orientation (i.e., shape) (9.4%, *SE* = 4.0)(*p* = 0.005). Consciousness did not interact with SOA/prime duration, *F*(1.62,24.25) = 2.66, *p* = 0.10, η^2^_p_ = .15.

In unmasked trials, the main effect for consciousness, *F*(1.15,17.31) = 12.11, *p* < 0.002, η^2^_p_ = .45, indicates that the participants were conscious of the orientation of the prime (67.4%, *SE* = 9.4) more often than they saw only a glimpse of something (28.1%, *SE* = 8.2) (*p* = 0.040) or were unaware of the presence of the prime (‘saw nothing’)(4.5%, *SE* = 1.1)(*p* < 0.001). The finding that the observers were not fully conscious of the presence or orientation of the prime in every trial can be explained by the low contrast (and the short 94 ms duration) of the primes.

In catch trials, the main effect for Consciousness (*F*(1.01,15.21) = 106.37, *p* < 0.001, η^2^_p_ = .88) showed that the participants were more often conscious of ‘nothing’ (83.6%, *SE* = 4.3), as compared with being conscious of ‘something’ (15.9%, *SE* = 4.1)(*p* < 0.001) or of the prime’s orientation (1.0%, *SE* = 0.5) (*p* < 0.001). Thus in 17% of trials the observers reported that they had seen at least ‘something’ presented. This reflects either liberal rating criteria (i.e., the observers were biased to report having seen ‘something’ rather than ‘nothing’) or it may have resulted from the flickering masks themselves sometimes eliciting an image about something else.

### Forced-choice response to prime’s orientation

We used R [33] and lme4 package (vers.1.1–7) [34] to perform a generalized (logit) mixed effect analysis of the proportion of correct forced-choice responses to the prime’s orientation in the critical CFS masked trials (Fig 2B). We used Consciousness and SOA as fixed factors and participant and the running number of the stimulus block as random factors, glmer(accuracy ~ SOA/prime duration * consciousness + (1|participant) + (1|block), family = binomial). *P*-values were calculated by Anova function in Car package (vers. 2.0–25). Because there is no standardized way to calculate the *p*-values for single-trial mixed-effect models, we computed also the 95% confidence intervals (CIs). The effect is considered statistically significant when the CI does not include zero.

Consciousness had a main effect, χ2^(1, N = 16)^ = 156.70, *p* < 0.001, 95% CI [0.235, 0.479], showing that the proportion of correct responses increased as a function of rated consciousness. In addition, SOA/prime duration interacted with Consciousness, χ2^(1, N = 16)^ = 16.21, *p* < 0.001 [0.095, 0.274], suggesting that accuracy increased as function of SOA/prime duration: accuracy increased in trials with the highest awareness rating (‘saw the orientation’), χ2^(1, N = 16)^ = 5.70, *p* < 0.017 [0.059, 0.617], but not in trials with ‘saw something’ rating, χ2^(1, N = 16)^ = 0.810, *p* = 0.368 [-0.063, 0.170], or ‘saw nothing’ rating’, χ2^(1, N = 16)^ = 0.94, *p* = 0.331 [-0.092, 0.031]. Importantly, in the trials with ‘saw nothing’ rating the accuracy level was .501 and it did not differ from the guessing rate [-0.043, 0.125]. In other words, the participants could not discriminate the orientation of the prime better than chance when they reported no awareness of the presence of the prime.

Next, we examined whether the orientation of the target (congruent vs. incongruent with that of the prime) influenced the discrimination of prime’s orientation. The participants discriminated in ‘saw nothing’ trials the prime’s orientation correctly in 66.4% of congruent trials and 33.8% in incongruent trials, *t*(15) = 4.03, *p* = 0.001 (average = 50.1%), suggesting that they were biased to follow the orientation of the target when they reported not having seen any prime and thus did not have any relevant information on which to base their judgements. To test whether this bias influenced the accuracy of prime discrimination, we performed signal detection analysis of the accuracy data by scoring correct responses in congruent trials as hits and incorrect trials in incongruent trials as false alarms. The bias free estimate of prime discrimination (*d*’) in ‘saw nothing’ trials was 0.028, which did not differ from the chance level of 0, *t*(15) = .65, *p* = 0.524 [-.344, 0.675]. Also Bayesian analysis with JASP 0.8.4 (JASP Team, https://jasp-stats.org) gave moderate support for the null hypothesis (i.e., chance level performance) (BF_01_ = 3.246). In ‘saw something’ trials, the accuracy percent in congruent (56.5%) and in incongruent (57.9%) trials did not differ from each other, *t*(14) = -0.20, *p* = 0.849 (average = 57.2%; one of the participants was excluded because there was not enough ‘saw something’ ratings to calculate the accuracy scores as a function of congruency). The bias free estimate of prime discrimination (*d*’) in ‘saw something’ trials was 0.407, which was higher than the chance level of 0, *t*(14) = 2.77, *p* = 0.015 [0.141, 1.321] and higher than the *d*’ in ‘saw nothing’ trials, *t*(14) = 2.408, p = 0.030 [0.059, 1.209].

The bias to respond according to target’s orientation was present when the participants reported that they had not seen any prime at all (*c* = -0.487), *t*(15) = -3.714, *p* = 0.002 [-1.558, -0.338]. When they reported having seen a glimpse of something, the bias (*c* = 0.019) was smaller than in ‘saw nothing’ trials (*t*(14 = 2.454, *p* = 0.028) and *c* did not differ from zero, which indicates neutral response criterion, *t*(14) = 0.186, *p* = 0.855 [-0.475, 0.573]. Bayesian analysis gave moderate support for the null hypothesis (i.e., lack of bias) in ‘saw something’ trials (BF_01_ = 3.753). In summary, the participants could not discriminate the orientation of the prime when they reported having not seen any prime, as indicated by the bias-free *d*’. In such trials, they did not have any conscious information about prime’s orientation, but because they were forced to choose one of the orientations, the orientation of the target could influence their forced-choice decisions. However, partial awareness of the prime (‘saw something’) increased the accuracy level above the chance level and the bias completely disappeared. This occurred in spite the fact, that the ‘saw something’ rating implicates that the participants were guessing and did not have any consciousness of the prime’s orientation. We conclude that the accuracy of forced-choice prime discrimination was not contaminated by the target’s orientation and that the subjective measure of awareness was exhaustive: when the participants reported that they could not see any prime at all, they could not discriminate prime’s orientation.

### Response times

The response times in the critical CFS masked conditions (Fig 2C) were first screened by eliminating trials with incorrect responses to the target or response times longer than 2s. After that, trials longer than 3 standard deviations from the experiment’s mean (> 1478 ms) were eliminated. Response times were log transformed for the analyses, but in Fig 2C we report the original, observed values, to help the interpretation. For analyzing the response time priming effects on single trial basis, we used R [33] and lme4 package (vers.1.1–7) [34] to perform a linear mixed effects analysis of the relationship between congruency, SOA/prime duration, and consciousness, which were entered as fixed effects. As random effects, we had intercepts for participants and for the running number of stimulus block: lmer(log(response time) ~ congruency * SOA/prime duration * Consciousness + (1|participant) + (1|block)). *P*-values and 95% confidence intervals were calculated.

Response times became longer as rated consciousness increased, χ2^(1, N = 16)^ = 110.72, *p* < 0.001 [-0.150, -0.009]. Priming was elicited, as indicated by the significant effect for Congruency, χ2^(1, N = 16)^ = 70.27, *p* < 0.001 [0.005, 0.050]. SOA/prime duration did not have a main effect on priming (SOA/prime duration x Congruency: χ2^(1, N = 16^ = 0.03, *p* = 0.8697 [-0.025, 0.009]). However, Consciousness interacted with Congruency, χ2^(1, N = 16)^ = 69.99, *p* < .001 [0.024, 0.085], suggesting that conscious trials were associated with larger priming than unconscious trials. In addition, the influence of consciousness on priming was marginally significantly modulated by SOA/prime duration (SOA/prime duration x Consciousness x Congruency: χ2^(1, N = 16)^ = 3.50, *p* = 0.0614 [-0.001, 0.044]). The failure of the 3-way interaction to reach statistical significance at the alpha level of 0.05 most probably was due to the small number of ‘saw orientation’ trials (see Fig 2A). To obtain a clearer view on the priming effects at each level of reported consciousness, we analyzed the congruency effects separately at each rating level as a function of SOA/prime duration.

Analysis of ‘saw nothing’ trials showed significant priming, χ2^(1, N = 16)^ = 10.80, *p* = 0.001 [0.002, 0.047]. The observed priming effects were on average 4 ms, 14 ms and 15 ms for SOAs of 94, 188, and 282 ms, respectively, but the size of priming did not depend on SOA/prime duration, χ2^(1, N = 16)^ = 0.020, *p* = 0.89 [-0.018, 0.016]. See Fig 2D for the modeled priming effects which show a flat SOA function. However, because the observed priming effects suggest that the congruency might have interacted with SOA/prime duration, we tested the interaction also by conducting Bayesian ANOVA on log RTs in ‘saw nothing’ trials with Congruency and SOA/prime duration as fixed factors and Participant and Block as random factors. It gave strong support favoring the null hypothesis for the interaction between congruency and SOA/prime duration, as compared with the model including only the main effects of SOA/prime duration and congruency (BF_10_ = 0.005). In other words, the data are 200 times more likely under the two main effects model than under the model that adds the interaction.

In ‘saw something’ trials, priming occurred, χ2^(1, N = 14)^ = 18.02, *p* = 0.001 [0.053, 0.124] and it depended on SOA/prime duration, χ2^(1, N = 14)^ = 7.63, *p* = 0.001 [-0.075, -0.013]: priming was suppressed at the longest SOA/prime duration.

Statistically significant priming occurred also when the participants reported that they ‘saw orientation’, χ2^(1, N = 14)^ = 130.98, *p* < 0.001 [0.060, 0.123]. The priming effects in ‘saw orientation’ increased as a function of SOA/prime duration. χ2^(1, N = 14)^ = 10.51, *p* = 0.001 [0.053, 0.189].

### Accuracy

The accuracy scores in CFS masked trials (Fig 2D) were analyzed with generalized (logit) linear mixed effects model of the lme4 package in R: glmer(accuracy ~ congruency * Consciousness + (1|participant), family = binomial). Unlike in the analysis of response times, the block and SOA/prime duration factors were not included as random factors, because the model failed to converge. The mean accuracy rates for congruent and incongruent targets were 98% and 96%, respectively, both in ‘saw nothing’ and ‘saw something’ trials. The corresponding values in ‘saw orientation’ trials were 99% and 94%. The congruency effect was statistically significant, χ2^(1, N = 16)^ = 27.00, *p* < 0.001 [-0.847, -0.219], indicating that priming occurred. Congruency interacted with Consciousness, χ2^(1, N = 16)^ = 5.03, *p* = 0.025 [-0.997, -0.073], showing that priming of response accuracy increased as a function of consciousness. This finding was due to larger priming in ‘saw orientation’ trials (5%) than in the ‘saw nothing’ (2%) and ‘saw something’ (2%) trials.

In general, the size of priming in accuracy was very small. The important point is that the incongruent trials were not associated with higher accuracy than the congruent ones, implying that the priming effects observed in the response times were not due to speed-accuracy trade-off.

## Discussion

The depth of suppression during continuous flash suppression (CFS) has been debated. It has been suggested that little or no processing occurs for stimuli whose visibility has been suppressed by CFS [27], whereas some studies have suggested that high level semantic processing of invisible stimuli can occur during CFS [13]. A recent review [35] on the depth of suppression induced by different masking techniques placed CFS on a relatively low level of the functional hierarchy of suppressive effects, between binocular-rivalry suppression and suppression by backward pattern or metacontrast masking. This placement was consistent with the result that response priming of shape survived metacontrast masking but not CFS [27]. The primary purpose of the present experiments was to test whether unconscious processing of shape, indexed with response priming, occurs when the conscious visibility of the prime is masked with CFS. In contrast to the previous study [27], we used abrupt prime onset and shorter SOAs/prime durations. Our experiment showed that response priming occurred when the participants reported not being conscious of the presence of the prime stimuli.

In previous experiments using metacontrast masking as the suppression method, the response priming effects have increased as a function of SOA [26]. In metacontrast masking, the visibility of the prime is masked by presenting the mask after the prime. The manipulation of SOA is typically made by keeping the duration of the prime constant, while manipulating the inter-stimulus interval between the offset of the prime and onset of the target, which influences the visibility of the prime depending on the relative energy of the prime and mask [3]. Vorberg et al. [26] manipulated the SOA between 14 and 84 ms. They observed a linear increase of the response priming effect, while discrimination of the prime remained at a chance level. However, the participants’ ability to detect the presence of the prime increased as a function of SOA and thus the primes were not completely invisible at the longer SOA. In the second experiment [26], discrimination of the prime followed u-shaped SOA function, first decreasing and then increasing, whereas the priming effect increased linearly as a function of SOA. In the present study, the SOAs/prime durations were longer and masking continued during the whole SOAs/prime duration and therefore the procedure is not comparable to those in Vorberg et al. [26]. The advantage of CFS masking is that the influence of prime’s duration or SOA on the time course of priming can be studied without confounding them with visibility. Our experiment showed that SOA/prime’s duration did not influence statistically significantly the priming effect when the observers did not report having any perception of the prime at all. Thus, the strength of unconscious priming stayed constant across the studied SOAs/prime durations. This pattern suggests that the response activation level triggered by invisible prime has an upper limit that cannot be exceeded with presenting the prime for longer durations. Once the activation has reached the limit, it does not continue to build up, unless the prime breaks the suppression and becomes consciously perceived. In addition, our results showed that the size of priming effect increased dramatically as a function of SOA/prime’s duration only when the observers reported having seen the orientation of the prime. This is logical, as when the SOA increases, more time for consciously guided behavior becomes available. By contrast, when the participants reported having seen only a glimpse of something (but not the orientation), the increase of SOA did not benefit priming as there was no relevant conscious information available concerning the orientation. In ‘saw something’ trials, the priming effect at the longest SOA/prime duration was reduced. A post-hoc explanation for this finding might state that that prolonged conscious processing of partial information inhibited the use orientation information in guiding speeded responses to targets (for inhibition of access to unconscious information by conscious processing, see [36]).The important point here is that partial awareness (of ‘something’) interfered rather than improved priming, suggesting that priming in ‘saw nothing’ trials, particularly at the longest SOA/prime duration, is very difficult to explain with partial awareness of the prime.

The accuracy of forced-choice response to the orientation of prime also depended on the rated level of consciousness. Accuracy in discriminating the prime’s orientation was the highest and improved as the SOA/prime duration increased in trials involving subjective awareness of the prime’s orientation. When ‘something’ was seen, the discrimination accuracy was lower than in the trials involving reported awareness of the orientation, and accuracy was not influenced by SOA/prime’s duration. This could be expected on the basis of the visibility reports which imply that the observers did not have any conscious orientation information which could have been used during the prolonged duration. Most importantly, in unconscious trials (‘nothing’ rating) performance was at chance level across the SOAs/prime durations, confirming that the observers did not have any task-relevant conscious information when they subjectively reported not having seen the prime. Null visibility corresponded to change performance, suggesting that the subjective reports were exhaustive. The rationale in the prime discrimination task was that a participant who is conscious of the minimal information needed for discriminating the orientation of the prime would be able to discriminate the prime’s orientation better than expected by chance. Thus, in ‘saw nothing’ trials the participants were not aware of the task-relevant aspects of the prime that are necessary to produce the priming effect. In spite of that, response priming was elicited. It is important to note that the ‘objective’ prime discrimination task measured access to task-relevant shape information. We did not have any ‘objective’ task for measuring access to lower-level visual features which would be indicative of observers’ consciousness of the presence of the prime. This may be important, because higher level unconscious processing of an image might be enabled by—or co-occur with—conscious access to some of its low-level features, even when these low-level features are not relevant to the processed dimension [11]. Gelbard-Sagiv et al. [11] showed that repetition priming of face identity under CFS did not occur when the observers were not conscious of any feature of the prime. Repetition priming occurred only when the observers were conscious of some non-task-relevant low-level feature of the prime (color or location). Thus, in our study it remains possible that the observers might have been conscious of some low-level feature of the prime in spite of reporting no awareness of the prime, and this may have enabled the unconscious priming of orientation. However, further studies are needed to test whether or not the findings of Gelbard-Sagiv et al. [11] will generalize from face perception to other types of task or from repetition priming to other types of priming.

The overall speed of responding to the target varied as a function of consciousness. In trials without reported consciousness of the presence of the prime (‘saw nothing’), response latencies were the fastest, whereas the latencies were the longest in the most fully conscious trials (‘saw orientation’). Thus, consciousness of the prime slowed down the overall speed of responses. Because the participants were required to respond to the target and to rate the consciousness of the prime as well as to make the forced-choice discrimination concerning prime’s orientation in each trial, it is plausible that when the prime entered consciousness, the prime and target were both processed by conscious attention, perhaps serially, which interfered with the speed of responding to the target. In trials without any consciousness of the prime, conscious attention could be focused solely on the target and responses to the targets were faster. In trials with partial consciousness of the prime (‘saw something’), the response speed increased as the SOA/prime duration became longer, perhaps because the participants were conscious of the prime’s presence and therefore attended to it for a prolonged time and tried to perceive its orientation. The differences in the general response speed clearly suggest that trials with different levels of rated consciousness were processed using different strategies.

We used a 3-point perceptual awareness scale instead of the typical 4-point PAS scale [28]. The lowest rating levels (‘saw nothing’, ‘saw glimpse of something’) corresponded to the original lowest PAS levels, whereas the highest level (‘I think/guess I saw the orientation’) replaced the two highest ones in the original scale (‘saw almost clearly’, ‘saw clearly’). Reducing the number of rating levels carries the risk that the primes with ‘saw nothing’ ratings are less unconsciously perceived as the primes with the same rating but in the context of a wider scale. We do not find any evidence in the present results suggesting that the lack of the ‘saw clearly’ alternative in the present study caused any problems for the validity of measuring conscious perception. Only in about 9% of the critical masked trials the participants rated that they thought or guessed they had seen the orientation of the prime. This low rate was expectable because the contrast of the stimulus was individually calibrated to a level where 50% or slightly less of the subjective responses were ‘saw nothing’. In our previous study using 4-point PAS [31], the contrast of Gabor patches was calibrated in detection task to a level in which about 50% of the stimuli received the lowest rating (‘saw nothing’). In the actual experiment, the distribution of ratings was such that ‘nothing’ was seen in 55% of trials, ‘something’ was seen in 40% of trials, ‘almost clear’ perception was present in 3% of trials, and ‘clear’ perception in 2% of trials. Most of the participants never used the highest rating. Inclusion of the 4-point scale in the present study hardly would have changed the distribution of ratings in the two lowest rating levels. It is worth noting that the PAS scale was created with [28] and often used in backward masking experiments that manipulate target’s duration [e.g., 37], which produces more variability in the subjective visibility because stimuli presented for a sufficiently long duration will inevitably be seen clearly.

Fang and He [38] found with functional magnetic resonance imaging (fMRI) that under inter-ocular suppression the visual stimuli activated areas in the dorsal stream but not in the ventral stream. After this finding, a common assumption has been that CFS interferes with processing in the ventral stream, so that stimuli under CFS are processed predominantly in the dorsal stream [18,19]. In principle, our findings are in line with the view that CFS suppresses primarily the processing of high spatial-frequencies by the ventral stream. This would account for the suppression of conscious vision, while processing in the dorsal stream remained relatively intact, which would explain the unconscious priming effects. However, recent brain imaging results have challenged the view that CFS affects dominantly the processing in the ventral stream (for review, see [39]). Hessellman and Malach’s [40] fMRI study used Mondrian masks and found that the suppressed stimuli elicited residual activation not only in the dorsal stream but also in the ventral stream. In addition, Sterzer, Haynes and Rees [41] suppressed the conscious visibility of pictures of faces and houses with Mondrian-like masks and could predict with above-chance accuracy the category of invisible stimuli on basis of the fMRI signal from high-level ventral visual cortex. In addition, multivariate pattern analysis indicated enhanced decoding of elongated tools both in the ventral and dorsal visual stream [42], contradicting the idea that dorsal "vision-for-action" processing is exclusively preserved under CFS.

Yuval-Greenberg and Heeger [43] found that CFS reduced activity in early visual cortex. The activation in V1 elicited by the lowest contrast stimulus did not differ from that elicited by catch trials; activation was reduced but present for higher contrast stimuli. The threshold for detecting the presence of the lowest contrast stimulus was determined so that the d’ was 0 (i.e., chance level), implying that the participants never were conscious of the lowest contrast stimulus. Thus, the suppression was stronger than in the present study and in Peremen and Lamy [27] in which some of primes were consciously perceived. Too strongly suppressed stimuli may be too low below the limen of consciousness to elicit any activity in early visual cortex. Yuval-Greenberg and Heeger [43] suggest that CFS does not switch neural activity “on” and “off,” which would correspond to the dichotomy between “aware” and “unaware” states, but it influences consciousness by modulating the contrast gain of neural responses. Thus, it is conceivable that the residual activations [41, 42], feeding either to ventral or dorsal streams, might mediate unconscious priming effects, provided the CFS masking is not too strong to completely suppress early neural activation or too weak to allow conscious perception.

Why did not Peremen and Lamy [27] find unconscious response priming in their CFS experiments? One potential explanation was that they used too long SOAs/prime durations (200, 250, 350, 450, 550, or 650 ms). A long prime duration may produce inhibition or habituation that leads to suppression of priming. Barbot and Kouider [10] showed that a long 1000 ms prime duration led to negative repetition priming effect (i.e., responses to primed targets were slowed down; see also [11]), whereas a short prime duration produced the typical facilitator effect. We observed unconscious response priming at SOAs/prime durations of 188 and 282 ms which roughly correspond to Peremen and Lamy’s shortest SOAs. This speaks against the explanation that the SOAs in Peremen and Lamy’s study were too long to enable short-lived priming effects. Other reasons for their failure to obtain unconscious priming may be the gradual ramping up of the prime’s contrast and the long response times to the targets. The overall speed of responding in Peremen and Lamy’s study was rather slow, even in unconscious trials (> 670 ms), whereas in our unconscious trials responses were substantially shorter (≈ 500 ms). Assuming that unconscious response priming is driven by fast, short-lived activation, the delayed responses, in combination with the gradual onset of the prime, may have made their procedure insensitive to the effects of the prime.

Finally, we must note the masking procedure in our study did not resemble the conventional CFS procedure used to achieve robust inter-ocular suppression. The masking in the shortest prime duration/SOA condition (94 ms) did not in a strict sense represent CFS masking. The mask was not continuously flashed to the opposite eye but appeared only once, simultaneously with the prime. This kind of inter-ocular masking, in which the stimulus and mask are briefly presented simultaneously to the opposite eyes, is called dichoptic masking [44]. It shares common inter-ocular suppression mechanisms with binocular rivalry [45]. At present, it is not clear whether there are any fundamental differences between dichoptic masking and CFS in their suppression mechanisms, except that during CFS the prolonged duration and depth of suppression is caused by the summation of the suppression due to multiple flashes of the masks [2]. The longest sequence of Mondrian masks in our experiments involved only 3 flashes of masks at 11 Hz. Tsuchiya et al. [2] showed that the depth of suppression with 3-flash CFS was substantially weaker than the conventional CFS with 5 or more flashes. A recent study [46] showed that suppression in CFS peaks at approximately 1 Hz, which is well below the rates (10 Hz or more) typically used in CFS studies. This low temporal frequency tuning function became more pronounced for high target spatial frequencies and increasing mask contrast. However, the present study did not aim to suppress the prime stimulus as strongly as possible, but to the extent that it is presented at the limen of consciousness so that unconscious and conscious priming could be measured while keeping the prime stimulus constant. In this context, we conclude that inter-ocular suppression can efficiently suppresses the task-relevant shape information from consciousness, but the unconscious shape information can still influence speeded responses to subsequently presented stimuli.

## Supporting information

S1 File Consciousness. Frequency of each rating category response as a function of SOA/prime duration in CFS masked trials, in unmasked trials and in catch trials.
(XLS)

S2 File Data from CFS masked trials. Single trial data used in the analyses of forced-choice responses to prime’s orientation, response times, and accuracy.
(XLSX)
